# THE ROLE OF GENDER IN FATHERS' AND MOTHERS' PREFERENCES FOR FUTURE ADULT CHILDREN CAREGIVERS

**DOI:** 10.1093/geroni/igac059.487

**Published:** 2022-12-20

**Authors:** Naomi Meinertz, Megan Gilligan, J Jill Suitor

**Affiliations:** Iowa State University, Fairfax, Iowa, United States; Iowa State University, Ames, Iowa, United States; Purdue University, West Lafayette, Indiana, United States

## Abstract

Previous caregiving research has focused on a single care recipient; however, no research has explored the potential simultaneous care needs of fathers and mothers in the same family. Drawing from gender role theory, we use qualitative data from 76 spousal dyads from the Within-Family Differences Study to compare fathers’ and mothers’ explanations of which adult child they prefer as their future caregiver. In 72% of families, fathers and mothers did not share the same care preferences suggesting that care preferences are dispersed among multiple adult children within the same family. In families in which fathers and mothers shared care preferences, 75% chose the same daughter. Additional findings showed that fathers’ explanations for a preferred caregiver aligned with mothers’ explanations; however, differences were identified based on children’s gender. These findings shed light on the similarities and differences in parental care preferences and underscore the influence of gender in family care networks.

